# Separating Law and Liberty

**DOI:** 10.1007/s10982-025-09541-8

**Published:** 2025-08-14

**Authors:** Lars J. K. Moen

**Affiliations:** Department of Philosophy, University of Vienna, Vienna, Austria

## Abstract

How is law related to liberty? For republicans, a just law constitutes people’s liberty. Another prominent view is that while the law is not necessary for freedom, legal constraints do not make individuals unfree when they protect their moral rights. In this paper, I reject these views and defend a purely negative conception of liberty according to which the law necessarily makes individuals unfree. I show how this means legal constraints deny individuals something valuable. But importantly, these constraints can still have a beneficial effect on negative freedom overall. By enabling us to consider how much freedom and unfreedom a constraint causes, this conception of freedom provides a better basis for evaluating legal constraints than do the two rival conceptions. It is therefore by taking the law to necessarily make people unfree in some respects that we can meaningfully evaluate different legal arrangements in terms of their effect on freedom.

## Introduction

How is law related to liberty?^1^ Republicans consider law constitutive of liberty. Freedom comes into existence only with a certain legal arrangement.^2^ Proponents of a rights-based account of liberty understand legal constraints as compatible with freedom as long as they protect individuals’ moral rights.^3^ Negative-freedom theorists, on the other hand, take any act that prevents someone from performing an action to make that person unfree. Law enforcement therefore necessarily makes individuals unfree, although it may also increase their freedom overall by motivating non-interfering behavior.^4^

In this paper, I consider these three prominent accounts of liberty and its relation to law. I identify common misunderstandings of the negative view, which is the view I also defend. I thus argue for separating law and liberty. I begin, in the next section, by contrasting the three accounts. I then, in Section III, demonstrate how a conception of freedom either constituted by or compatible with legal constraints must be based on moral evaluation of these constraints. Republican freedom and rights-based freedom are therefore, in this sense, “moralized.”

Moralization is meant to ensure the value of liberty. On the rights-based view, the law is said to take away nothing valuable when it denies individuals opportunities to violate each other’s moral rights. We therefore need an account of moral rights before we can define freedom as something valuable. And for republicans, legal constraints deny citizens nothing valuable when they prevent them from acting contrary to their shared, or common, interests. Indeed, these constraints are themselves considered valuable because they provide citizens the protection they need to lead dignified, flourishing lives. On this view, then, we need a normative theory identifying the common interests before we can define freedom as something valuable.

In Sections IV and V, I reject these reasons for regarding certain legal constraints as compatible with freedom. While the constraints may be justified, they still take away something valuable. The crucial mistake made by defenders of both the rights-based and republican conceptions is their failure to assess the value of a freedom to do some action, *x*, independently of the value of doing *x*. By keeping freedom and action separate, we shall see that even a freedom to do an immoral action will typically be valuable despite the immoral action being valueless. Trusting someone with the freedom to do wrong is dignifying and important for personal autonomy. It can even make moral behavior more, not less, likely. We will see how this means republicans’ own concerns with trust and autonomy are incompatible with their view of constraints as themselves valuable.

Only pure negative liberty captures the fundamental liberal view that any constraint on what an individual can do takes away something valuable and therefore stands in need of justification. But endorsing this conception of freedom does not commit us to the view that such constraints are never justified. In fact, as I show in Section VI, legal constraints can have a positive effect on citizens’ pure negative liberty. By reducing liberty in some areas, they can enhance it in others. This is especially so when the law restricts people from denying each other what Feinberg calls “fecund options,” which are opportunities connected to many other opportunities.^5^ The law can thus, overall, be enabling rather than restricting.

It is only by defining liberty as necessarily conflicting with legal constraints that we can make such insightful evaluations of the law’s overall effect on liberty. And we shall see how it helps us understand and evaluate the constraints defended in the libertarian and republican theories underpinning rights-based and republican conceptions of freedom, respectively. Somewhat paradoxically, then, it is a non-moralized conception of liberty, which treats any legal constraint as a source of unfreedom, that best enables us to evaluate and justify legal constraints on the basis of liberty.

## Law and liberty

Pettit asks whether coercive law reduces the freedom of those subject to it and says “[t]he answer that appears to have a nearly universal hold on the minds of legal theorists and philosophers today is that yes, coercive law does always reduce people’s freedom.”^6^ This statement is highly inaccurate since many theorists, including Pettit himself, take liberty to depend on law in one shape or another. Republicans think of the law as constitutive of liberty.^7^ And many liberals and libertarians argue that enforcing just laws cannot make citizens unfree, though they disagree about what laws are just.^8^ Even those Pettit primarily aims his criticism at deny that coercive law necessarily reduces people’s freedom. Negative-freedom theorists hold that legal constraints deny individuals liberties they otherwise would have had, but law enforcement can discourage interference in ways resulting in a net increase of their overall liberty.^9^

On this purely negative account, an agent, A, is made unfree to perform some action, *x*, by some other agent, B, if and only if B makes A physically unable to do *x*. The view is purely negative as it does not matter why B acts toward A in a preventive manner. Nor does it matter whether the prevention is intentional, significant, or permissible.^10^ B prevents A from doing *x* either by using physical force to disable A from doing *x*, or by being disposed to use physical force so that B will successfully stop A from doing *x* if A tries to do *x*.^11^ Either way, B makes A unable and therefore unfree to do *x*.

Law enforcement will therefore deny an individual a negative freedom to do some criminal act, *x*, by taking an anticipatory preventive measure denying them the opportunity to do *x*.^12^ But more commonly, successful law enforcement does not deny people the freedom to do *x* but rather the freedoms to do certain actions in conjunction with doing *x*.^13^ That is, if you do *x*, you will be punished, and the punishment involves a loss of certain freedoms.

But while legal constraints thus make individuals unfree to perform certain actions, or combinations of actions, they need not reduce their overall freedom. By deterring people from interfering with one another and by enabling coordination that produces opportunities that otherwise would not have been possible, the law can expand citizens’ overall negative freedom.^14^ But since the extent to which the law has this desirable effect is an empirical issue, there is no conceptual connection between law and liberty. In an ideal anarchy, where such coordination and non-interfering behavior would occur without law enforcement, there would be more freedom than in any society with coercive law.^15^ However, the large-scale coordination modern societies depend on seems highly unlikely, if not impossible, without law. Realistically, therefore, law is crucial for promoting pure negative freedom in a modern society.

On other accounts of liberty, however, legal constraints need not be a source of unfreedom at all. On one such account, law protecting individuals’ moral rights makes no one unfree. On this rights-based account of liberty, you can be free to perform only actions you have a moral right to perform. Bader, in his recent account of rights-based freedom, takes these moral rights to include both Hohfeldian claim-rights and liberties.^16^ Actions you have a moral right, and can therefore be considered free, to perform therefore include actions you have a claim against others preventing you from performing and actions no one has a moral right to prevent you from performing.^17^ Actions you have no moral right, thus understood, to perform are actions you are not free to perform. These actions are outside of the scope of rights-based freedom.^18^ Since you cannot be free to perform these actions, you are not made unfree when someone prevents you from performing them. This rights-based view of freedom is particularly associated with libertarianism,^19^ but it can also be found in the works of some high liberals.^20^

What is most important for present purposes is that a rights-based account of freedom allows us to say that legal constraints not violating anyone’s moral rights make no one unfree. Law enforcement preventing A from performing an action A has no moral right to perform does not make A unfree because A was never free to perform such an action. And by performing such an action, A may be punished, even imprisoned, without a loss of liberty. While anyone has a moral right not to be confined to a prison cell, A forfeits this right by acting in a way they have no moral right to act. When A is then put in a prison cell and prevented from leaving, A is not made unfree to leave because they cannot be considered free to leave.^21^ On the pure negative account, on the other hand, A is made unfree simply because they are prevented from leaving the cell. It does not matter why they are so prevented. No matter how justified it might be, the prevention is a source of unfreedom equal to any other kind of prevention.

Finally, republicans defend the strongest connection between law and liberty. On their view of freedom as non-domination, legal restrictions are not only potentially compatible with freedom, freedom cannot exist without them. For Pettit, “the properly constituted law is constitutive of liberty.”^22^ Sellers, similarly, holds that there can be “no liberty without the law.”^23^ A “republican legal system,” he says, “secures ‘liberty’.”^24^ And Lovett thinks only the law can regulate “the use of coercive force in a manner consistent with our freedom.”^25^ These theorists share the republican view that to be free is to be independent from the arbitrary will of another agent. And only the law is thought to give citizens this independence. List also notes this link between the rule of law and republican freedom.^26^

The requirements for the rule of law are important for the republican connection between law and liberty.^27^ These requirements include that laws be predictable, clear, publicly known, consistent, and stable. They must be made under open, stable, clear, and general rules. They must also be interpreted by an independent judiciary, courts must be unbiased and accessible, and the police’s law enforcement must be effective. The clarity and predictability of what the law demands ensures that citizens know what compliance involves, and they can expect each other to comply. Republicans therefore consider the law essential for reducing uncertainty in individuals’ lives. A key aspect of republican freedom is to be protected by law so as to be able to plan ahead without feeling anxious about unpredictable interference in their lives.^28^

However, the law can also be used for oppressive, malevolent purposes.^29^ It ought to, but need not, be just. This is the widely accepted “fallibility thesis.”^30^ Republicans also accept it, and they therefore acknowledge that while freedom requires the rule of law, the rule of law does not entail freedom.^31^ The law must specifically promote citizens’ shared, or common, interests. On the modern, neo-republican interpretation, these are interests in a protected ability to exercise the basic liberties.^32^ A prisoner imprisoned under a law promoting common interests, thus understood, is therefore not considered unfree.^33^

Republican freedom also requires that the law be made and enforced under popular control. This requires various institutions, including a virtuous citizenry ready to contest legislation and law enforcement perceived to conflict with common interests.^34^ What exactly popular control demands of citizens is left vague, however, and indeed intentionally so by Pettit, as it is said to depend on local circumstances.^35^ But however virtuous citizens may be, popular control will realistically be limited.^36^ My focus here, however, is on the uncontroversial observation that republican freedom presupposes a well-functioning legal system.^37^

Republican freedom is clearly different from the negative and rights-based accounts of liberty introduced above. Republicans have mostly aimed their criticism at negative freedom.^38^ This view of freedom as the absence of prevention, they argue, ignores the significance of institutional protection against interference. A slave, for example, can be considered free, in the negative sense, to perform whatever actions no one prevents them from performing.^39^ Republicans consider this unacceptable, as they see slavery as the epitome of unfreedom.^40^ No one at the mercy of the arbitrary will of another, as a slave is in relation to their master, can be considered free in any way whatsoever.

The law ensures such independence by forcefully denying individuals opportunities to gain arbitrary power over another. Pettit acknowledges that “the law necessarily involves interference,” but interference serving common interests and protecting citizens against dependence on others, he says, “is not going to be arbitrary.”^41^ Such interference therefore makes no one unfree. “There will be systems of law available, at least in principle,” Pettit says, “which are entirely undominating and entirely consistent with freedom.”^42^ And Sellers notes that with a “shared commitment to the common good,” citizens can agree on a law that makes none of them unfree.^43^

## Moralizing freedom

An important step in evaluating these conceptions of liberty is to show that the rights-based and republican conceptions are moralized. Bader explains that moralization of freedom restricts either the *y*- or the *z*-parameter in MacCallum’s view of freedom as a triadic formula.^44^ For MacCallum, any conception of liberty involves an agent, *x*, who is free from some constraint, *y*, to do, or become, something, *z*. We restrict the *y*-parameter by saying that some constraints do not make you unfree because they are justified. Alternatively, we limit the *z*-parameter by identifying certain actions individuals can be neither free nor unfree to perform, because these actions are morally impermissible.

Pure negative freedom is not moralized in either of these ways, since it takes any physical prevention by another agent to make you unfree, however justified the prevention might be.^45^ And any action is an action you can be made unfree to do. It does not matter how permissible, or impermissible, the action might be. It therefore invariably treats legal constraints, like any other kind of constraint, as a source of unfreedom. This makes pure negative freedom what Carter calls a “value-free” concept, since it describes a state of affairs, or a relation between agents, without evaluating it.^46^ But it is not fully “value-neutral,” in Carter’s sense, as its normative significance will not be apparent from all ethical points of view.^47^ This significance concerns the ways negative freedom can be considered valuable, which is an important issue I turn to later in the paper.

Rights-based freedom, on the other hand, restricts both the *y*- and the *z*-parameter.^48^ Only actions you have a moral right to perform are included in the *z*-parameter, which means only these actions are actions you can be free or unfree to perform. Other actions are actions you are neither free nor unfree to perform. The justly imprisoned prisoner has forfeited their moral right not to be confined to a prison cell and is therefore neither free nor unfree to leave the cell. And constraints on behavior conflicting with others’ moral rights are excluded from the *y*-parameter. So, legal constraints on such behavior do not make you unfree, since you were never free to do these actions in the first place. The constraints on the justly imprisoned prisoner do not make the prisoner unfree because these constraints have been excluded from the set of constraints that can make the prisoner unfree.

Republicans connect freedom to law by excluding a kind of constraint from the *y*-parameter—that is, from the set of constraints that can make you unfree. As we have seen, legal constraints under popular control considered to serve common interests are not regarded as a source of unfreedom. The importance of constraints is more emphasized than on a rights-based conception, as constraints are considered necessary for ensuring popular control under which no one possesses the unchecked power to act contrary to common interests. As virtually all commentators on republican freedom observe, this makes for a moralized conception of freedom.^49^ The interference republicans deem compatible with freedom is interference justified based on moral considerations. A person justifiably forced to pay their taxes and the justly imprisoned prisoner are both constrained, and only by moralizing freedom can republicans say these justified constraints do not make these individuals unfree.

The often-observed problem with moralizing freedom is that by basing the definition of freedom on a normative theory—such as one defining moral rights or common interests—freedom itself cannot serve as a standard of evaluation independent of the theory.^50^ To perform a useful function in constructing the theory, freedom must be defined prior to the theory. This problem with moralization is recognized by at least some moralizers. Bader agrees that a moral theory is needed for identifying the moral rights that then determine what one can be considered free or unfree to do. Moralization, he admits, “excludes the notion of liberty from playing a fundamental justificatory role.”^51^

Pettit also acknowledges this problem, but he peculiarly denies that republican freedom is moralized.^52^ This issue is cleared up, however, when we notice that Pettit means something else by moralization than other contributors to the freedom literature do. Pettit first argues that republican freedom cannot be moralized because the issue of whether someone is subject to the uncontrolled power of another is factual, not evaluative.^53^ But this tells us nothing about whether the freedom concept is moralized or not. What matters is whether the concept is value-free, in the sense described above, not whether we can empirically assess someone’s freedom after freedom has been defined. We can define a rights-based account of freedom and then say it is a factual matter whether someone’s moral rights are respected and is therefore free. But the conception of freedom is still moralized because it is defined in terms of how it is permissible to treat someone. As for republican freedom, identifying which constraints make you unfree and which do not is an evaluative exercise concerning what constraints are justifiable. The conception of freedom is therefore moralized and not value-free. This view is not challenged by our ability to observe, as a matter of fact, whether someone is or is not free in this sense.

Pettit also suggests republican freedom cannot be moralized because it is neutral between conceptions of the good and imposes no comprehensive moral doctrine on anyone. It also conflicts with paternalism, he says.^54^ But while anti-paternalism and neutrality between conceptions of the good are opposed to “moralizing” in a certain sense, Pettit’s reason for thinking institutional constraints serving these values cannot make people unfree is that they are justified according to his normative theory. He thus takes unjustified constraints to make people unfree and justified constraints to not make people unfree. His republican conception of freedom is therefore moralized, in the relevant sense, and not value-free.^55^

## The value of liberty

But moralizers can accept that a moralized conception of liberty cannot serve a fundamental purpose in the formulation of a normative theory, and then try to argue that moralization is necessary for ensuring the value of freedom. We have to exclude justified constraints from the *y*-parameter, or impermissible actions from the *z*-parameter, they might say, in order to have a conception of freedom as something valuable. This is to say that freedom cannot be valuable if we conceptualize it as value-free and non-moralized. Bader explicitly endorses this line of reasoning.^56^

On the pure negative view, any prevention makes you unfree. It thus implies that you can be free, or unfree, to do morally impermissible actions, such as murdering or torturing someone, just as you can be free or unfree to do permissible actions. These freedoms are not valuable, the moralizer argues. When the law denies us such freedoms, it therefore takes away nothing valuable. So, to ensure the value of freedom, such legal interference cannot be understood to compromise individuals’ freedom. Legal restrictions denying individuals opportunities to do wrongful acts deny them nothing valuable. In Bader’s view, there is “no presumption in favor of letting people engage in such behavior.” But there are, he says, “plenty of reasons to prevent people from performing these actions. Interferences with such actions thus cannot constitute constraints on freedom.”^57^ Dworkin, similarly, denies that freedom can be valuable if we think of it as mere absence of constraint. To be valuable, he says, freedom should capture only “the ways in which we believe people ought to be free.”^58^ You should not, then, be considered unfree when you are prevented from infringing on another’s moral rights, since doing so is not a valuable act.

Republicans value life in a well-ordered republic under popular control and defend constraints necessary for maintaining such a society. Life in a political society is “a historical necessity,” Pettit says, and people must accept that realizing this necessity restricts what opportunities they have.^59^ Pettit labels interests in pursuing selfish ends as “irrelevant.”^60^ Society needs law, and the law must bind everyone. We have no reason to give any concern to interests people might have in behaving contrary to this ideal.^61^ Sellers, similarly, says that defining liberty in a non-moralized way as “pure absence of restraints lowers its value as a social ideal. Liberty stops being a status to be sought and becomes a retained privilege, perhaps too often granted or properly withheld.”^62^

Rights-based and republican accounts of freedom thus imply that freedom only has, what Carter calls, “specific” value, and not “non-specific” value.^63^ That is, only certain freedoms are considered valuable because of their particular content, which is what they are freedoms to do. Freedom as such is not considered valuable—that is, freedom has no value independently of its content. On the rights-based account, only freedoms to act in accordance with individuals’ moral rights are valuable. On the republican account, only freedoms to act in accordance with common interests are valuable. On either view, then, legal constraints can secure what is valuable without taking away anything of value insofar as they only deny individuals freedoms regarded as valueless.

Separating law and liberty therefore depends on a demonstration of why this picture is wrong; why even liberties taken away by a just law are valuable. I do so by showing how the rejection of freedom’s non-specific value rests on a failure to make two important distinctions. The first is the distinction between performing an action, *x*, and being free to do *x*. You can be in a position where no one will prevent you from doing *x* if you try, and thus be free to do *x*, without actually taking this opportunity to do *x*.^64^ Moralizers of freedom miss this distinction by maintaining that if doing *x* is not valuable, then being free to do *x* is not valuable. It may be wrong and valueless to act in ways that compromise another’s moral rights or conflict with common interests, but being free to do so is a different thing that may still be valuable. A can be free to violate B’s moral right, but as long as A does not act on this freedom, they will not harm B. And the freedom—the lack of constraint—can be valuable to A. And merely being free to act contrary to common interests does nothing to compromise common interests.

This points to a general problem with moralizing the *z*-parameter. On such a view, as we have seen, impermissible actions are excluded from the domain of freedom because they are considered valueless. For Bader and Nozick, they compromise moral rights. And for Dworkin, they conflict with his ideal of liberty as living in an ideal society where everyone enjoys equal concern and respect.^65^ But being free to act in such impermissible ways does not conflict with these ideals. Any freedom is compatible with respecting moral rights and with Dworkin’s ideal egalitarian society. It is only when the freedom is exercised that the ideal can be compromised. Bader suggests we define freedom’s *z*-parameter by including only those non-moralized freedoms that are universalizable—that is, freedoms people can have without denying anyone else a freedom.^66^ But by appreciating the distinction between freedom and action, we see that any freedom is universalizable. Generally, two or more people can each be free to occupy exactly the same physical space at exactly the same time. Only when one of them exercises this freedom do the others become unfree. So, while we may say that people should act so as to respect others’ moral rights, it does not follow that we should deny them freedoms not to do so. The problem may not apply to the republican moralizing of the *y*-parameter, however, as I shall return to in the next section.

Failing to distinguish between doing *x* and being free to do *x* leads moralizers to miss also the distinction between the value of doing *x* and the value of being free to do *x*. This is an important but commonly neglected distinction, as Feinberg and Kramer point out.^67^ The value of being free to do *x* is not wholly independent of the value of doing *x*, since it is likely to be greater if the value of doing *x* is of high value. But if doing *x* has negative value, it does not follow that the freedom to do *x* also has negative value. And if doing *x* is wrong, the value of being free to do *x* can still be positive. Cohen expresses this view when he says citizens can meaningfully oppose restrictions on overseas travel even if they have no desire to travel overseas.^68^ And Kramer does so by noting that while self-disembowelment is harmful and valueless, the freedom to disembowel oneself is valuable.^69^ Being trusted with the freedom to impose harm on oneself or others is valuable because it means being respected as a mature person capable of doing the right thing in the absence of external constraints.^70^

Law enforcement typically also shows individuals such trust, as it usually involves punishing after a crime has been committed. It thus denies no one the freedom to break the law. Anticipatory preventive measures are sometimes taken, but they can be unacceptably intrusive. Being constantly prevented from doing even something as serious as murder would make the law an unbearable presence in our lives. So, for this reason, people should be free even to commit serious crimes, at least in the absence of a good reason for expecting them to actually do so.

Being respected as a person capable of distinguishing right from wrong and acting morally in the absence of external constraints is dignifying and important for a sense of self-respect. By allowing individuals freedoms to perform only valuable actions, we do not express respect for them as people capable of making good decisions.^71^ Without a range of opportunities to reflect on, people will not feel in control of which opportunities to take and are then unlikely to develop a sense of dignity and self-respect.^72^ The interest in liberty, as Feinberg points out, is therefore not just about being free to do what you want or ought to do, but also in having other opportunities so as to be able to choose.^73^ Having a variety of opportunities to reflect on is important for developing the value of autonomy, independently of the value of the specific freedoms to do what you want or ought to do.^74^ We should therefore account for freedom’s non-specific value.^75^ Not only freedoms to perform morally permissible actions are valuable, as moralizers claim, since freedom as such has value independently of its particular content.

Bader, however, quickly dismisses the idea of freedom having non-specific value by saying the arguments for this view at best only suggest that any freedom may have instrumental value “contingent and dependent on particular empirical circumstances.”^76^ For any freedom, on this view, there is a possible situation in which it is valuable because it can be used for something valuable. The freedom to torture a baby may be valuable under very specific and desperate circumstances, for example. But Bader here again fails to distinguish between doing an action and being free to do an action. The value of performing an action like torturing a baby may be valuable only under very specific circumstances. But it does not follow that the value of being free to perform an action that only rarely has value is “dependent on particular empirical circumstances.”

## The value of constraint

Republicans, however, offer a different challenge to the view that freedom has non-specific value. As we have seen, republicans consider certain constraints not just compatible with but constitutive of freedom. These are the constraints deemed necessary for protecting everyone against others interfering with them in ways conflicting with common interests. And this seems to mean that someone merely having the freedom to do something impermissible is problematic, whether they exercise the freedom or not. Unless people are constrained from acting against common interests, people will be dependent on each other’s goodwill, which means they cannot be regarded as free persons.

For republicans, the law is crucial for protecting people against ending up in such precarious positions. Legal protection is considered essential to liberty even if people are not inclined to interfere with each other. In Kant’s Kingdom of Ends, everyone is morally committed to treating each other respectfully as an end in oneself. But even though no one acts in a way republicans disapprove of, Pettit says no one in the Kingdom enjoys the legal protection needed for being independent of the will of another.^77^ So, without legal protection, no one is free.

We may question whether the law can actually ensure this independence, since people will still be dependent on the goodwill and virtue of the government officials providing and maintaining the legal protection.^78^ But the relevant point here is that by taking this view, republicans deny not just the value of impermissible behavior but also the value of being free to behave in such ways. Or if law enforcement cannot prevent the impermissible action, republicans at least deny the value of being free to act in this way without appropriate legal punishment.^79^ The freedoms denied by just law enforcement are not even considered valuable to the person constrained. Pettit thinks interests in freedom to act against common interests are “irrelevant,” and Skinner considers such interests “a failure of rationality.”^80^ Nothing of value is therefore lost when someone is denied opportunities to act in such a way. By thus regarding certain constraints as valuable, and not just saying the actions they prevent are valueless, republicans can offer a different defense of the view that freedom only has specific value, and therefore a different argument for moralizing liberty, than that we find in rights-based accounts of freedom.

But when we consider the arguments for freedom’s non-specific value identified in the previous section, we see that this attempt to connect law and liberty comes at a high cost contemporary republicans are unwilling to accept. For Pettit, a free person enjoys self-respect and the ability to relate to anyone as their equal. And we have seen that a sense of one’s own dignity and standing depends on freedom to reflect on various opportunities, some of which it will be morally wrong to take. Enjoying the dignified status of republican freedom, at least on its contemporary liberal understanding, therefore involves being trusted with a degree of freedom as such.^81^ This observation conflicts with the republican emphasis on denying individuals’ freedoms not considered valuable. Republicans cannot both demand protective constraints that deny people freedoms considered valueless and idealize the self-respect that requires that people have such freedoms. Developing the autonomy and self-respect republicans value depends on opportunities to make unconstrained, dignified choices between doing right or wrong. Since self-respect is considered a key part of the common interests republican institutions promote, the republican concern with common interests restricts the extent to which republicans can demand legal constraints.^82^

Relying on legal constraints rather than virtue also expresses a lack of trust. In a large society, the law will likely be important for coordinating individuals’ behavior and for providing them with an assurance of each other’s compliance with social norms and conventions. But by regarding legal constraints as necessary even if only to add another layer of protection against behavior conflicting with common interests, republicans express a lack of trust in individuals’ inclination to do the right thing in the absence of such constraints. And this can be counter-productive, as an expanding body of behavioral studies suggests.^83^ Legal constraints can impair people’s sense of self-determination and make them feel less responsible for their behavior. This can consequently make them less inclined to behave virtuously.^84^ This is not always the case, and sometimes external constraints are advisable, but making people feel trusted with the freedom to do wrong can make them more likely to do what is right.

Pettit also accepts that trust can have this positive effect.^85^ People want others to think well of them and will therefore not take advantage of their trust.^86^ Trust can therefore make government officials more inclined to serve common interests. But to trust someone to do the right thing means giving them the freedom not to do so. Pettit also admits that relying on trust and virtue “offer[s] less satisfying images of solidity and security.”^87^ Valuing trust therefore implies valuing freedoms to perform valueless actions. Insofar as republicans rely on trust, then, they accept freedom’s non-specific value. And they then rely not on law but on people’s goodwill toward each other. Pettit indeed says that “[p]eople must be willing to accept the fact of often having to be vulnerable to others, and often having to trust themselves to the civility of those others not to do them harm.”^88^

Republican freedom, then, is the status one enjoys under institutions satisfying a normative theory that balances various moral concerns. And among those concerns, at least on the contemporary interpretation of Pettit and others, pure negative freedom is important. Republicans must therefore also accept that legal constraints deny citizens something valuable, however justified the constraints may be all things considered.

## Freedom-promoting law

Neither the rights-based nor the republican view of freedom therefore implies a rejection of pure negative freedom. In the different moral evaluations on which the rights-based and republican conceptions of freedom are based, pure negative freedom plays a significant role in the ways identified in the last two sections. Pure negative freedom plays a part in the evaluations of legal constraints on which the rights-based and republican conceptions are based. In this final section, I consider further how it performs this more fundamental role, and I show why it is therefore more useful for evaluating legal constraints. It is because pure negative freedom implies that legal constraints necessarily take away some of citizens’ freedom that it enables us to consider how these constraints can enhance their freedom by facilitating respect and cooperation among citizens and preventing obstructive behavior. A freedom concept in one way conflicting with law is therefore best equipped for justifying it.

Republicans, Pettit says, reject the view that “while the law coerces people and thereby reduces their liberty, it compensates for the damage done by preventing more interference than it represents.”^89^ They instead “hold that the properly constituted law is constitutive of liberty in a way that undermines any such talk of compensation.” But this denies liberty any place in the evaluation of the law and relegates it to a mere label we put on the legal status we think citizens ought to have after we have concluded this evaluation. Taking instead the view Pettit here rejects enables us to use liberty in our assessments of legal constraints. It allows us to see, first, that the law takes away freedoms, or, more commonly, combinations of conjunctively exercisable freedoms, and therefore something valuable. But we can then compare this loss of liberty to the law’s positive effects on overall freedom. Freedom thus plays a constructive role in the evaluation. We use it to consider what is the just legal arrangement, rather than taking it merely to refer to the status one holds under such an arrangement.

While freedom has non-specific value, the law can be justified in denying citizens freedoms because of the loss of liberty likely to occur if we do not deter citizens from behaving toward each other in various wrongful ways. The law, while interfering with citizens, can therefore make them freer overall. “Contemplating criminal legislation,” Feinberg says, “always involves appraisals of the ‘trade-off’ between diminished political liberty and enlarged *de facto* freedom.”^90^ When the law prevents harm to others, he says, “most of us *usually* make a gain in *de facto* freedom that more than compensates us for any loss of liberty to engage in the proscribed conduct.”^91^ Feinberg especially points out that law can ensure people “fecund options,” which are options that “lead to many further options.”^92^ By protecting these options, the law can therefore ensure individuals more freedoms than it denies. Of course, some legal restrictions are not justified based on liberty, as they “open nowhere near as many or as fecund options as they close.”^93^ Included among such unjustified restrictions are the constraints we in the last section saw that republicans are unwilling to defend, even though they would make people less dependent on each other’s goodwill.

Now, rights-based freedom can play some role in evaluating law, because it does not treat a just law as constitutive of freedom, but rather as potentially compatible with freedom. Law can be justified, all things considered, but still conflict with rights-based freedom. As Bader notes, while there is always a pro tanto reason against infringing on an individual’s moral rights, there will be cases in which such interference is justified.^94^ Nozick also refrains from firmly denying that infringements on moral rights are never justifiable.^95^ A libertarian might maintain a weighty commitment to respecting rights-based freedom but allow that freedom may be compromised when conflicting concerns are particularly weighty.^96^ Freedom, thus understood, is therefore just one concern, albeit an especially important one, to be taken into account in evaluations of law. It is not as fundamental as pure negative freedom, because it is based on a moral theory identifying moral rights in which pure negative freedom has a place, as demonstrated above. But rights-based freedom is not based on a theory of just and legitimate institutions but rather a constraint to be accounted for in such a theory.

In such theorizing, it is therefore more useful than republican freedom, since the latter is the status one enjoys under just and legitimate institutions. Your republican freedom can remain intact despite your being interfered with and disadvantaged in ways one might understand to infringe on your moral rights, as long as this is considered to serve common interests. A just law will not systematically target particular groups or individuals for such treatment, but sometimes someone has to suffer, and this is merely “tough luck,” Pettit says, and denies that it makes the victim unfree.^97^ This type of case seems to be what Bader has in mind when he says moral rights may sometimes be justifiably infringed on. But he can use his rights-based conception of freedom to show that there is a normative concern against it.

A core argument in this paper is that the non-moralized, purely negative conception of liberty enables us to best capture the value of not being denied opportunities. And this is an important concern to be taken into account when we evaluate legal constraints on individuals, since any such constraint then stands in need of justification. Pure negative freedom concept is value-free, in the sense explained above, as it describes a social relation between agents without implying anything regarding its permissibility or justification. Because it is itself not evaluative in this sense, we can meaningfully use it to evaluate and compare different normative theories in terms of how they promote or respect individuals’ liberty.^98^

Cohen (1979) takes this strategy when he criticizes Nozick’s (1974) libertarianism for defending not freedom from interference as such, but only freedom from interference incompatible with Nozick’s entitlement theory of justice.^99^ In this theory, people are not made unfree when they are prevented from using what Nozick considers to be others’ justly acquired private property, or when prevented from performing various actions as punishment for having violated another’s property right.^100^ But this means these people are made unable to do various actions because of the protection of others’ property. Property owners, or those protecting their property rights, thus indisputably make others unfree, in the negative, non-moralized sense, and thus deny them something valuable. Libertarians like Nozick therefore face the burden of demonstrating why such interference is nonetheless justifiable.

With a view of liberty constituted by a just law, republicans cannot pose such a challenge to libertarians. Pettit is critical of a libertarian, minimal state that cannot do enough, as he sees it, to provide citizens with the protection and resources they need to enjoy the status of republican freedom.^101^ This, then, becomes a debate about whether a just law is one that staunchly protects the moral rights libertarians take individuals to have, or one that will occasionally allow for interference libertarians disapprove of in order to ensure that all citizens have what they need to enjoy a certain standing in their society. The two parties’ moralized conceptions of freedom will not help in advancing this debate since they merely express these two opposing positions. Libertarians can say that republicans show insufficient respect for individuals’ moral rights, and republicans can deny the existence of such rights and consider the interference justified, perhaps because redistribution is necessary for ensuring everyone’s dignity and self-respect.

With a non-moralized conception of liberty, the two can more meaningfully criticize each other’s commitment to liberty. In particular, republicans can follow a strategy like Cohen’s by saying that respecting the moral rights libertarians take individuals to have will have bad consequences for liberty, either for society as a whole or for certain groups in society, presumably those with less private property. They can thus see taxation and restrictions on private property rights as liberty-enhancing measures, which in turn can ensure citizens the liberty they need to enjoy the dignified standing republicans defend.

Such a constructive appeal to liberty in a defense of interfering legal measures is possible only with a non-moralized conception of liberty that is itself defined independently of any justification of legal constraints. It is thus, somewhat paradoxically, a conception of freedom that treats any legal constraint as a source of unfreedom that is most useful for evaluating and justifying such constraints.

## Footnotes

1 Some philosophers distinguish between liberty and freedom. See, for example, Ronald Dworkin, *Justice for Hedgehogs* (Cambridge, MA: Belknap Press of Harvard University Press, 2011), pp. 366–367; Bernard Williams, ‘From Freedom to Liberty’, *Philosophy and Public Affairs* 30, no. 1 (2001): 3–26. I shall take the more conventional approach of treating the two terms synonymously.

2 Frank Lovett, ‘A Republican Argument for the Rule of Law’, *Critical Review of International Social and Political Philosophy* 26, no. 2 (2023): 137–158; Philip Pettit, *Republicanism: A Theory of Freedom and Government* (Oxford: Oxford University Press, 1997); Philip Pettit, *On the People’s Terms: A Republican Theory and Model of Democracy* (Cambridge: Cambridge University Press, 2012); Mortimer Sellers, ‘Republicanism, Liberalism, and the Law’, *Kentucky Law Journal* 86, no. 1 (1997): 1–30.

3 For a libertarian account of rights-based liberty, see Robert Nozick, *Anarchy, State, and Utopia* (New York: Basic Books, 1974). For a liberal account, see Ronald Dworkin, ‘What Is Equality? Part 3: The Place of Liberty’, *Iowa Law Review* 73, no. 1 (1987): 1–54.

4 Ian Carter, *A Measure of Freedom* (Oxford: Oxford University Press, 1999); Matthew H. Kramer, *The Quality of Freedom* (Oxford: Oxford University Press, 2003).

5 Joel Feinberg, ‘The Interest in Liberty on the Scales’, in *Values and Morals: Essays in Honor of William Frankena, Charles Stevenson, and Richard Brandt*, ed. Alvin Goldman and Jaegwon Kim (Boston: Reidel, 1978), pp. 21–35.

6 Philip Pettit, ‘Law and Liberty’, in *Legal Republicanism: National and International Perspectives*, eds. Samantha Besson and José Luis Martí (Oxford: Oxford University Press, 2009), p. 39.

7 Lovett, ‘A Republican Argument for the Rule of Law’, pp. 137–158; Pettit, ‘Law and Liberty’; Sellers, ‘Republicanism, Liberalism, and the Law’.

8 Ralf M. Bader, ‘Moralizing Liberty’, in *Oxford Studies in Political Philosophy*, vol. 4*,* eds. David Sobel, Peter Vallentyne, and Steven Wall (Oxford: Oxford University Press, 2018), pp. 141–166; Dworkin, ‘What Is Equality?’; Nozick, *Anarchy, State, and Utopia*.

9 Carter, *A Measure of Freedom*; Kramer, *The Quality of Freedom*.

10 Hillel Steiner, *An Essay on Rights* (Oxford: Blackwell, 1994), pp. 9–12, 17–21.

11 Steiner, *An Essay on Rights*, pp. 33–41.

12 Matthew H. Kramer, ‘Why Freedoms Do Not Exist by Degrees’, *Political Studies* 50, no. 2 (2002): 230–243, p. 233.

13 Steiner, *An Essay on Rights*, ch. 2.

14 Steiner (*An Essay on Rights*) defends the “law of conservation of liberty,” according to which the total freedom in a society is fixed and cannot be increased. On this view, then, the law can neither increase nor decrease overall liberty, though it can produce a more desirable distribution of liberty in the population. This “law,” however, is widely rejected among negative-liberty theorists. For persuasive critiques, see Ian Carter, ‘Respect for Persons and the Interest in Freedom’, in *Hillel Steiner and the Anatomy of Justice: Themes and Challenges*, eds. Stephen de Wijze, Matthew H. Kramer, and Ian Carter (London: Routledge, 2009), pp. 167–184; Kramer, *The Quality of Freedom*, pp. 209–240.

15 Carter, *A Measure of Freedom*, pp. 265–267; Michael Taylor, *Community, Anarchy and Liberty* (Cambridge: Cambridge University Press, 1982), ch. 4.

16 Bader, ‘Moralizing Liberty’, p. 141, fn. 1.

17 For a recent discussion of the distinction between claim-rights and liberties, see Matthew H. Kramer, *Rights and Right-Holding: A Philosophical Investigation* (Oxford: Oxford University Press, 2024), ch. 2.

18 On the “scope” of different conceptions of liberty, see Lars J. K. Moen, ‘Freedom and Its Unavoidable Trade-Off’, *Analytic Philosophy* 65, no. 1 (2024): 22–36.

19 It is standardly associated with Nozick, but we find similar rights-based views of liberty in the works of other influential libertarians, such as Eric Mack, ‘Moral Individualism and Libertarian Theory’, in *Liberty for the 21st Century*, ed. Tibor R. Machan and Douglas B. Rasmussen (Lanham: Rowman & Littlefield, 1995), p. 57; Murray N. Rothbard, *For a New Liberty: The Libertarian Manifesto* (New York: Macmillan, 1973), p. 43.

20 Dworkin, ‘What Is Equality?’.

21 Bader, ‘Moralizing Liberty’, pp. 152–153.

22 Pettit, *Republicanism*, p. 35

23 Sellers, ‘Republicanism, Liberalism, and the Law’, p. 30.

24 Sellers, ‘Republicanism, Liberalism, and the Law’, pp. 2–3.

25 Lovett, ‘A Republican Argument for the Rule of Law’, p. 154.

26 Christian List, ‘Republican Freedom and the Rule of Law’, *Politics, Philosophy & Economics* 5, no. 2 (2006): 201–220.

27 These requirements are influentially outlined by Raz and later more elaborately by Kramer. See Joseph Raz, *The Authority of Law: Essays on Law and Morality* (Oxford: Oxford University Press, 1979), pp. 214–218; Matthew H. Kramer, *Objectivity and the Rule of Law* (Cambridge: Cambridge University Press), ch. 2.

28 Pettit, *Republicanism*, p. 85.

29 Raz, *The Authority of Law*, pp. 211–214.

30 Leslie Green, ‘Legal Positivism’ *The Stanford Encyclopedia of Philosophy*, ed. Edward N. Zalta (2018), accessed 25 May 2024. URL: https://plato.stanford.edu/archives/spr2018/entries/legal-positivism.

31 Frank Lovett, *A General Theory of Domination and Justice* (Oxford: Oxford University Press, 2010), pp. 111–119; Pettit, *Republicanism*, pp. 5, 36–37, 84.

32 Pettit, *On the People’s Terms*.

33 But Pettit (*Republicanism*, p. 26, fn. 1) says this prisoner is made “non-free.” He also says a just constraint “conditions” a person’s freedom, but it does not “compromise” it (*Republicanism*, pp. 76–77).

34 Frank Lovett, *The Well-Ordered Republic* (Oxford: Oxford University Press, 2022), p. 208; Pettit, *Republicanism*, p. 266.

35 Pettit, *On the People’s Terms*, p. 32, fn. 8, p. 68, fn. 38.

36 Assaf Sharon, ‘Domination and the Rule of Law’, in *Oxford Studies in Political Philosophy*, eds. Peter Vallentyne, David Sobel, and Steven Wall (Oxford: Oxford University Press, 2016), pp. 128–155.

37 Frye argues that social norms can by themselves provide the protection republican freedom requires. The other republicans mentioned here, however, deny that social norms alone can provide the impartial rule enforcement required for protecting people from dependence on another’s arbitrary will. But the important point for this paper is that interfering institutions, which will at least realistically include legal institutions, are considered constitutive of republican freedom. See Harrison P. Frye, ‘Freedom without Law’, *Politics, Philosophy & Economics* 17, no. 3 (2018): 298–316. For another recent discussion of the institutional requirements of republican freedom, see Lars J. K. Moen, *The Republican Dilemma: Promoting Freedom in a Modern Society* (Oxford: Oxford University Press, 2024), ch. 5.

38 Philip Pettit, ‘Republican Freedom: Three Axioms, Four Theorems’, in *Republicanism and Political Theory*, eds. Cécile Laborde and John Maynor (Oxford: Blackwell, 2008), pp. 102–130; Philip Pettit, ‘The Instability of Freedom as Noninterference: The Case of Isaiah Berlin’, *Ethics* 121, no. 4 (2011): 693–716; Quentin Skinner, ‘Freedom as the Absence of Arbitrary Power’, in *Republicanism and Political Theory*, eds. Cécile Laborde and John Maynor (Oxford: Blackwell, 2008), pp. 83–101.

39 Some negative-freedom theorists take freedom to perform an action to require the ability to do it, and not just the absence of another agent’s prevention. See, for example, G. A. Cohen, ‘Two Addenda to “Freedom and Money”’, in *On the Currency of Egalitarian Justice and Other Essays in Political Philosophy*, ed. Michael Otsuka (Princeton: Princeton University Press, 2011), pp. 193–199; Kramer, *The Quality of Freedom*.

40 Lovett, *The Well-Ordered Republic*, pp. 36–38; Pettit, *Republicanism*, p. 22; Skinner ‘Freedom as the Absence of Arbitrary Power’.

41 Pettit, *Republicanism*, pp. 36–37.

42 Pettit, *Republicanism*, p. 66.

43 Sellers, ‘Republicanism, Liberalism, and the Law’, p. 26.

44 Bader, ‘Moralizing Liberty’; Ralf M. Bader, ‘Moralized Conceptions of Liberty’, in *The Oxford Handbook of Freedom*, eds. David Schmidtz and Carmen E. Pavel (New York: Oxford University Press), pp. 59–75; Gerald C. MacCallum, ‘Negative and Positive Freedom’, *Philosophical Review* 76, no. 3 (1967): 312–334.

45 As Waldron notes, moralization takes freedom ‘in the direction of a positive definition’. See Jeremy Waldron, ‘Homelessness and the Issue of Freedom’, *UCLA Law Review* 39, no. 1 (1991): 295–324, p. 307.

46 Ian Carter, ‘Value-Freeness and Value-Neutrality in the Analysis of Political Concepts’, in *Oxford Studies in Political Philosophy*, vol. 1, eds. David Sobel, Peter Vallentyne, and Steven Wall (Oxford: Oxford University Press), 279–304.

47 Carter’s distinction between “value-free” and “value-neutral” mirrors Kramer’s distinction between “value-neutral” and “value-independent,” respectively. See Matthew H. Kramer, ‘Conceptual Analysis and Distributive Justice’, in *The Oxford Handbook of Distributive Justice*, ed. Serena Olsaretti (Oxford: Oxford University Press, 2018), pp. 367–385.

48 Bader, ‘Moralizing Liberty’; Bader, ‘Moralized Conceptions of Liberty’.

49 Ian Carter. ‘How Are Power and Unfreedom Related?’, in *Republicanism and Political Theory*, eds. Cécile Laborde and John Maynor (Oxford: Blackwell, 2008), pp. 64–65; John Christman, ‘Review of *Republicanism: A Theory of Freedom and Government* by Philip Pettit’, *Ethics* 109, no. 1 (1998): 202–206; David Estlund, ‘Review of *On the People’s Terms: A Republican Theory and Model of Democracy*, by Philip Pettit’, *Australasian Journal of Philosophy* 92, no. 4 (2014): 799–802; Matthew H. Kramer, ‘Liberty and Domination’, in *Republicanism and Political Theory*, eds. Cécile Laborde and John Maynor (Oxford: Blackwell 2008), p. 41; Christian List and Laura Valentini, ‘Freedom as Independence’ *Ethics* 126, no. 4 (2016): 1043–1074, pp. 1058–1066; Christopher McMahon, ‘The Indeterminacy of Republican Policy’, *Philosophy & Public Affairs* 33, no. 1 (2005): 67–93, pp. 69–70; Lars J. K. Moen, ‘Republicanism and Moralised Freedom’, *Politics, Philosophy & Economics* 22, no. 4 (2023): 423–440; Jeremy Waldron, ‘Pettit’s Molecule’, in *Common Minds: Themes from the Philosophy of Philip Pettit*, ed. Robert Goodin, Geoffrey Brennan, Frank Jackson, and Michael Smith (Oxford: Clarendon, 2007), pp. 151–154.

50 Carter, *A Measure of Freedom*, p. 72; G. A. Cohen, ‘Freedom, Justice and Capitalism’, *New Left Review* 126 (1981): 3–16, p. 11; G. A. Cohen, *Self-Ownership, Freedom, and Equality* (Cambridge: Cambridge University Press), p. 62; Kramer, *The Quality of Freedom*, pp. 102–103; Steiner, *An Essay on Rights*, p. 15.

51 Bader, ‘Moralizing Liberty’, p. 158, fn. 36.

52 Pettit, ‘Law and Liberty’, p. 40.

53 Philip Pettit, ‘The Determinacy of Republican Policy: A Reply to McMahon’, *Philosophy & Public Affairs* 34, no. 3 (2006): 275–283, pp. 278–280; Pettit, ‘Republican Freedom’, p. 117; Pettit, ‘Law and Liberty’, p 40; Pettit, *On the People’s Terms*, p. 58.

54 Pettit, ‘Republican Freedom’, pp. 127–128, n. 19; Pettit, *On the People’s Terms*, pp. 58–59.

55 For further elaboration on how republican freedom is moralized, see Moen, ‘Republicanism and Moralised Freedom’.

56 Bader, ‘Moralizing Liberty’; Bader, ‘Moralized Conceptions of Liberty’.

57 Bader, ‘Moralized Conceptions of Liberty’, p. 71.

58 Dworkin, ‘What Is Equality?’, p. 5.

59 Pettit, *On the People’s Terms*, p. 167.

60 Philip Pettit, ‘The Common Good’, in *Justice and Democracy: Essays for Brian Barry*, eds. Robert E. Goodin, Keith Dowding, and Carole Pateman (Cambridge: Cambridge University Press, 2004), p. 152.

61 Pettit, *Republicanism*, p. 93; Pettit, *On the People’s Terms*, p. 167.

62 Sellers, ‘Republicanism, Liberalism, and the Law’, p. 11.

63 Carter, *A Measure of Freedom*. Kramer (*The Quality of Freedom*) uses the terms “content-dependent” and “content-independent” value instead of “specific” and “non-specific” value, respectively.

64 Taylor, *Community, Anarchy and Liberty*, pp. 153–154. On Kramer’s trivalence account, this distinction looks different. On that view, freedom requires not just the absence of agential prevention but also the ability to perform the action. See Kramer, *The Quality of Freedom*, pp. 212–213.

65 Dworkin, ‘What Is Equality?’.

66 Bader, ‘Moralizing Liberty’, p. 164.

67 Feinberg, ‘The Interest in Liberty on the Scales’, p. 27; Kramer, *The Quality of Freedom*, pp. 240–245.

68 G. A. Cohen, *History, Labour, and Freedom: Themes from Marx* (Oxford: Oxford University Press), p. 271, fn. 23.

69 Kramer, *The Quality of Freedom*, pp. 241–242.

70 There may be special cases, however, where denying someone a freedom is not, at least to some people, to deny them something valuable. Kramer (*The Quality of Freedom*, pp. 444–445) uses the example of putting up a fence along a path to protect people from falling down a steep stony bank. Most people, at least, will not consider the opportunity to fall down the bank valuable.

71 Carter ‘Respect for Persons and the Interest in Freedom’, pp. 175–177.

72 Carter (*A Measure of Freedom*, p. 60) considers freedom as such constitutive of self-respect. Van Hees objects and says this value is not constitutive but instrumental. For present purposes, however, it suffices to say that freedom as such is valuable. I need not go into how it is valuable. Showing people respect involves allowing them a variety of opportunities to act in different ways, some of which will be valuable, others will not be. See Martin van Hees, *Legal Reductionism and Freedom* (Dordrecht: Kluwer, 2000), pp. 154–156.

73 Feinberg, ‘The Interest in Liberty on the Scales’, pp. 31–32.

74 Thomas Hurka, ‘Why Value Autonomy?’, *Social Theory and Practice* 13, no. 3 (1987): 361–382. Kramer criticizes Raz for failing to see this when he maintains that “autonomy is valuable only if it is directed at the good.” See Matthew H. Kramer, *Liberalism with Excellence* (Oxford: Oxford University Press, 2017), pp. 233–236; Joseph Raz, *The Morality of Freedom* (Oxford: Oxford University Press, 1986), p. 411.

75 Carter, *A Measure of Freedom*, ch. 2; Kramer, *The Quality of Freedom*, pp. 242–245; Van Hees, *Legal Reductionism and Freedom*, ch. 8.

76 Bader, ‘Moralizing Liberty’, p. 162, fn. 42

77 Pettit, *On the People’s Terms*, pp. 181–184.

78 Matthew H. Kramer, ‘Freedom and the Rule of Law’, *Alabama Law Review* 61, no. 4 (2010): 827–845.

79 Pettit, *Republicanism*, p. 22; Quentin Skinner, *Liberty before Liberalism* (Cambridge: Cambridge University Press, 1998), p. 72.

80 Pettit, ‘The Common Good,’ p. 152; Quentin Skinner, ‘The Republican Ideal of Political Liberty’, in *Machiavelli and Republicanism*, ed. Gisela Bock, Quentin Skinner, and Maurizio Viroli (Cambridge: Cambridge University Press, 1990), p. 304. This connection between personal and common interests is central in the republican tradition (e.g., Marcus Tullius Cicero, *On Obligations*, trans. P. G. Walsh (Oxford: Oxford University Press, 2008), pp. 7–8).

81 Pettit (*On the People’s Terms*, p. 11, fn. 8) himself notes that his theory may be appropriately labelled “republican liberalism or liberal republicanism.”

82 A firmer commitment to constraints at the expense of negative liberty requires republicans to weaken their liberal commitments. See Lars J. K. Moen, ‘Republican Freedom and Liberal Neutrality’, *Journal of Ethics and Social Philosophy* 26, no. 2 (2023): 325–348.

83 E.g., Samuel Bowles, *The Moral Economy: Why Good Incentives Are No Substitute for Good Citizens* (New Haven and London: Yale University Press, 2016); Bruno S. Frey, *Not Just for the Money: An Economic Theory of Personal Motivation* (Cheltenham: Edward Elgar, 1997).

84 Edward L. Deci, Richard Koestner, and Richard M. Ryan, ‘A Meta-Analytic Review of Experiments Examining the Effects of Extrinsic Rewards on Intrinsic Motivation’, *Psychological Bulletin* 125, no. 6 (1999): 627–668.

85 Philip Pettit, ‘The Cunning of Trust’, *Philosophy & Public Affairs* 24, no. 3 (1995): 202–225; Pettit, *Republicanism*, pp. 268–269.

86 See also Geoffrey Brennan and Philip Pettit, *The Economy of Esteem: An Essay on Civil and Political Society* (New York: Oxford University Press, 2004).

87 Pettit, *Republicanism*, p. 266.

88 Pettit, *Republicanism*, p. 266.

89 Pettit, *Republicanism*, p. 35.

90 Feinberg, ‘The Interest in Liberty on the Scales’, p. 33.

91 To be precise, the law can be said to take away a deontic freedom, or a permission. But as already noted, it typically does not deny the physical freedom to do what it prohibits. Punishing this action instead involves reducing the number of combinations of freedoms one can exercise in conjunction with the prohibited action.

92 Feinberg, ‘The Interest in Liberty on the Scales’, p. 28.

93 Feinberg, ‘The Interest in Liberty on the Scales’, p. 35, n. 13.

94 Bader, ‘Moralizing Liberty’, p. 159.

95 Nozick, *Anarchy, State, and Utopia*, p. 30, fn.

96 Kramer criticizes this form of “threshold deontology” for failing to grasp the absoluteness of moral obligations. See Matthew H. Kramer, *Torture and Moral Integrity: A Philosophical Investigation* (Oxford: Oxford University Press, 2014), ch. 1.

97 Pettit, *On the People’s Terms*, pp. 175–179.

98 Felix Oppenheim, *Political Concepts: A Reconstruction* (Chicago: University of Chicago Press, 1981), p. 154.

99 G. A. Cohen, ‘Capitalism, Freedom and the Proletariat’, in *The Idea of Freedom: Essays in Honour of Isaiah Berlin*, ed. Alan Ryan (Oxford: Oxford University Press, 1979), pp. 9–25.

100 Nozick, *Anarchy, State, and Utopia*.

101 Pettit, *On the People’s Terms*, pp. 10–11.

